# Cumulative effect of reproductive factors on ideal cardiovascular health in postmenopausal women: a cross-sectional study in central south China

**DOI:** 10.1186/s12872-015-0172-4

**Published:** 2015-12-21

**Authors:** Xia Cao, Jiansong Zhou, Hong Yuan, Zhiheng Chen

**Affiliations:** Department of Health Management Center, the Third Xiangya Hospital, Central South University, Tongzipo Road 138, Changsha, Hunan Province 410013 China; Mental Health Institute, the Second Xiangya Hospital, the Central South University, Changsha, Hunan Province China; Department of Clinical Pharmacology Center, the Third Xiangya Hospital, the Central South University, Changsha, Hunan Province China

**Keywords:** Cardiovascular health, Reproductive risk, Menopause, Chinese women

## Abstract

**Background:**

The American Heart Association developed the Life’s Simple 7 metric for defining cardiovascular health. Little is known, however, whether co-occurring reproductive factors, which affects endogenous oestrogen levels during a woman’s life, also influences ideal cardiovascular health in postmenopausal women.

**Methods:**

Using data on a cross-sectional study with a convenience sample of 1,625 postmenopausal women (median age, 60.0 years) in a medical health checkup program at a general hospital in central south China 2013–2014, we examined the association between cumulative reproductive risk and ideal cardiovascular health in postmenopausal women. A cumulative risk score (range 0 to 4) was created by summing four reproductive risk factors (age at menarche, age at menopause, number of children, and pregnancy losses) present in each individual from binary variables in which 0 stands for favorable and 1 for less-than-favorable level. Ideal levels for each component in Life’s Simple 7 (blood pressure, cholesterol, glucose, BMI, smoking, physical activity, and diet) were used to create an ideal Life’s Simple 7 score [0–1 (low), 2, 3, 4, 5 and 6–7 (high)].

**Results:**

Participants with earlier age at menarche (odds ratio [OR] =0.42 [95 % CI 0.26-0.48]), earlier age at menopause [0.46 (0.32-0.58)], who have more than three children (0.42 [0.38-0.56]) and have history of pregnancy losses [0.76 (0.66-0.92)] were more likely to attain low (0–1) ideal Life’s Simple 7 after adjustment for age. Participants were more likely to attain low (0–1) ideal Life’s Simple 7 as exposure to the number of reproductive risk factors increased [OR (95 % CI) of 0.52 (0.42-0.66), 0.22 (0.16-0.26), and 0.16 (0.12-0.22) for cumulative reproductive risk scores of 1, 2, and 3 or 4, respectively, each versus 0].

**Conclusions:**

The postmenopausal Chinese women with an increasing number of reproductive risk factors were progressively less likely to attain ideal levels of cardiovascular health factors.

## Background

The American Heart Association (AHA) has defined the construct of “ideal cardiovascular health” based on 7 cardiovascular health metrics to measure progress toward their 2020 Impact Goal [1]. A substantial body of evidence demonstrates that the ideal cardiovascular health index is associated with reduced cardiovascular morbidity and mortality [2, 3].

Known as the “change of life”, menopause is one of the important stages of a woman’s life with the loss of ovarian function and reproductive determinants of health have essentially formed in this stage. It is well known that postmenopausal women have a high risk of developing cardiovascular disease [4, 5]. However, the postmenopausal women possibly still benefit from the endogenous oestrogen produced during their reproductive history, due to the effects it exerted on conventional cardiovascular risk factors [6]. Among female reproductive factors, early menarche and early menopause have been identified to be associated with the risk of mortality from cardiovascular disease in postmenopausal women [7–9]. Nevertheless, there remains less knowledge on whether reproductive factors, developing during a woman’s reproductive span, would have a protective role in good cardiovascular health after menopause. Moreover, so far, studies examining the relationship between reproductive factors and cardiovascular risk factors have seldom explored their cumulative effect, that is whether exposure to multiple reproductive factors at the same time might influence the favorable cardiovascular health profile.

Accordingly, the aim of the present study was to explore the association between cumulative reproductive risk and ideal cardiovascular health using data from a sample of urban postmenopausal women in central south China who participated in the 2013–2014 Changsha Women’s Health Screening Program (CSWHSP).

## Methods

### Study participants

CSWHSP is a follow-up study assessing risk factors underlying women’s reproductive and cardio-metabolic diseases sponsored by Changsha Labor and Social Security Bureau started in 2011. Participants of the present study were 1,927 community postmenopausal female residents who entered this program during 2013 to 2014 conducted by the health management center of Third Xiangya Hospital of Central South University. Menopausal status was determined by self-reported menstrual cycle characteristics. Postmenopausal women had gone 1 year or longer without menses according to STRAW criteria as well [10]. We excluded participants from the study who had prevalent cardiovascular diseases (defined as a self-reported history of stroke, myocardial infarction or heart failure). Moreover, those who had missing data for one or more of the anthropometric variables under study were also excluded. Therefore, the final analysis set consisted of a total of 1,625 participants. The study followed the standards of the Declaration of Helsinki and has been approved by the Institutional Review Board of the Third Xiangya Hospital, Central South University. As the dataset used in this study is consisted of de-identified data from a retrospective cohort, the written informed consents from the participants who receiving physical check-up were waived by the approval of the IRB.

### Data collection

#### Reproductive risk factors

We assessed 4 reproductive factors that have been proposed as mainly components of female reproductive history in previous literature [6, 7]. The four reproductive factors of interest was self-reported through the questionnaire and included age at menarche, age at menopause, number of children, and pregnancy losses. We created a sum of the reproductive risk factors present in each individual from binary variables in which 0 stands for favorable and 1 for less-than-favorable level. The cut-off points were based on previous evidence and theoretical knowledge. Earlier age at menarche was defined as starting menarche at an age < 12 years [11]. Earlier age at menopause was defined as either natural menopause or surgical removal of ovaries at an age < 46 years [12]. High parity was defined as the number of times a woman has borne children ≥ 3 [13]. Pregnancy losses were defined as having history of miscarriage, abortion or stillbirth [14].

#### Ideal cardiovascular health index

The ideal cardiovascular health index comprised 7 ideal metrics was adapted from the AHA metric [1]. Each metric of cardiovascular health including cigarette smoking, body mass index (BMI), physical activity, diet, total cholesterol (TC), blood pressure (BP), and fasting blood glucose (FBG) were classified into three levels (poor = 1 points; intermediate = 2 point; ideal = 3 points; total score: 7–21 points). Cigarette smoking status, physical activity, and diet were self-reported through the questionnaire collected in person by trained doctors. Trained staff reviewed the completed questionnaires and entered the responses into a database.

Detailed definitions of “poor”, “intermediate”, and “ideal” levels for all seven metrics are given below. Cigarette smoking: ideal, never smoker; intermediate, smoking cessation; poor, current smoker. BMI: ideal, <25 kg/m^2^; intermediate, 25–30 kg/m^2^; poor, ≥30 kg/m^2^. We had insufficient information about physical activity and dietary intake. Physical activity was assessed using the questionnaire that assesses the frequency and duration of participation in moderate and vigorous physical activity during the past 30 days. Physical activity: ideal, ≥90 min/week; intermediate, 0–89 min/week; poor, never exercise. Ideal diet included following 4 components: ≥450 g/d fruits or vegetables, ≥2 servings of fish per week, 3 or more 1-oz servings a day of whole grains, and sodium < 1500 mg/d. Diet categories were as follows: ideal, having ≥ 3 ideal diet components of the above 4 components; intermediate, having 1–2 ideal diet components; poor, having none ideal diet components. TC: ideal, untreated TC < 5.17 mmol/L; intermediate, untreated TC 5.18-6.19 mmol/L or treated TC < 5.17 mmol/L; poor, TC ≥ 6.20 mmol/L. BP: ideal, untreated systolic BP (SBP) < 120 mmHg and diastolic BP (DBP) < 80 mmHg; intermediate, SBP 120-139 mmHg or DBP 80–89 mmHg or treated BP < 120/80 mmHg; poor: SBP ≥ 140 mmHg or DBP ≥ 90 mmHg. FBG: ideal, untreated FBG < 5.6 mmol/L; intermediate, FBG 5.6-6.9 mmol/L or treated FBG < 5.6 mmol/L; poor, FBG ≥ 7.0 mmol/L. Current use of medications was based on self-report.

#### Clinical measurement of cardiovascular risk factors

All examinations were done at Third Xiangya Hospital of Central South University. Body weight and height were measured by a balance scale, with the participant standing erect, arms at her side and feet together. Weight was measured to the nearest 0.1 kg. Height was measured to the nearest 0.5 cm. Body mass index (BMI, kg/m^2^) was defined as the weight in kilograms divided by the square of the height in meters. Three blood pressure readings were taken after a quiet rest in a sitting position for 5 min, with the average used for data analysis.

Blood samples were drawn from the antecubital vein by careful venipuncture using a 21-G sterile syringe without stasis at 08:00–10:00 hours after a fasting period of 12 h and analyzed using standard protocols. Concentrations of fasting blood glucose (FBG), total cholesterol (TC), and triglycerides (TG) were determined by enzymatic colorimetric assay.

### Statistical analyses

Statistics analysis was calculated using SPSS statistical software package version 19.0 (SPSS Inc., Chicago, IL, USA). Age- adjusted odds ratios (ORs) and 95 % confidence intervals (CIs) of each of Life’s Simple 7 component (poor, intermediate, and ideal) were calculated for each reproductive risk factor (earlier age at menarche, earlier age at menopause, more than three children, and pregnancy losses) using multinomial logistic regression. Multinomial logistic regression was used to estimate age- adjusted ORs and 95 % CIs for the associations between individual reproductive risk factors and ideal Life’s Simple 7 scores (2, 3, 4, 5, and 6–7 versus 0–1). Age- adjusted odds ratios and 95 % CIs for higher ideal Life’s Simple 7 scores (2, 3, 4, 5, and 6–7 versus 0–1) by cumulative reproductive risk score (1, 2, and 3 or 4 each versus 0) were also estimated using multinomial logistic regression. We hypothesized the cumulative reproductive risk score was associated with ideal Life’s Simple 7 scores independent of each individual reproductive risk factor. To test it, we constructed models that included a continuous variable for cumulative reproductive risk score and each individual reproductive risk factor, and assessed whether the variable for cumulative reproductive risk remained statistically significant. Statistical significance was considered at *P* < 0.05 and all tests were two-sided.

## Results

A total of 1,927 postmenopausal women participated in the 2013–2014 CSWHSP, from whom 302 were excluded: 48 with history of stroke, 76 with history of myocardial infarction, 2 with history of both stroke and myocardial infarction, and 81 lacking information regarding the seven metrics. We further excluded participants with missing data on the age at menarche (*n* = 67) and age at menopause (*n* = 28). After these exclusions, a final sample of 1,625 (84.3 % of the eligible sample) participants was included in our analysis. Those excluded due to missing data were more likely to have high education (92.1 % vs. 89.3 %), but have a comparable median Life’s Simple 7 total score [17(15–20) vs. 17(14–20)].

The characteristics of participants are shown in Table 1. The median age of participants was 60.0 years (25th-75th percentiles: 54.0-65.0 years). The percentage of the women with reproductive risk factors ranged from 7.3 % (with more than three children) to 63.9 % (pregnancy losses). A total of 53.8 % of participants reported one reproductive risk factor; 19.3 % reported two; and 2.1 % reported three or four reproductive risk factors. Meanwhile 3.4 % of the participants had 0–1 ideal health metrics and 7.6 % of participants had 6–7 ideal health metrics.

**Table 1:** Characteristics of the postmenopausal women in CSWHSP 2013–2014 without prevalent cardiovascular disease (*N* = 1625)

Characteristics	
Age, median (25th-75th percentiles) years	60.0(54.0-65.0)
Education level: college graduate or advanced, n (%)	1451(89.3 %)
Individual reproductive risk factors	
Earlier age at menarche, n (%)	239(14.7)
Earlier age at menopause, n (%)	206(12.7)
With more than three children, n (%)	119(7.32)
Pregnancy losses, n (%)	1038(63.9)
Number of reproductive risk factors	
0, n (%)	404(24.9)
1, n (%)	874(53.8)
2, n (%)	313(19.3)
3 or 4, n (%)	34(2.1)
Ideal health metrics	
BMI < 25 kg/m^2^, n (%)	1140(70.2)
Physical activity ≥ 90 min/week, n (%)	735(45.2)
Healthy dietary intake, n (%)	186(11.4)
Never smoker, n (%)	1621(99.8)
Untreated total cholesterol < 5.17 mmol/L, n (%)	671(41.3)
Untreated blood pressure < 120/80 mm Hg, n (%)	539(33.2)
Untreated fasting blood glucose < 5.6 mmol/L, n (%)	1093(67.3)
Ideal Life’s Simple 7 score categories	
0-1, n (%)	56(3.4)
2, n (%)	235(14.5)
3, n (%)	433(26.6)
4, n (%)	480(29.5)
5, n (%)	298(18.3)
6-7, n (%)	123(7.6)
Life’s Simple 7 score (7–21), median (interquartile range)	17(14–20)

After age- adjustment, participants with earlier age at menarche were less likely to have ideal levels of BMI, physical activity, blood pressure, and FBG (Table 2). Participants with earlier age at menopause were less likely to have ideal levels for BMI, physical activity, and FBG (each *P* < 0.01). Participants with high parity were less likely to have ideal levels for BMI and physical activity but more likely to have ideal levels of cholesterol. Participants with history of pregnancy losses were less likely to have ideal levels for FBG.

**Table 2:** Odds ratios for Life’s Simple 7 components associated with individual reproductive risk factors among CSWHSP 2013–2014 participants^a^

Components	Earlier age at menarche	Earlier age at menopause	With more than three children	Pregnancy losses
	Odds ratio (95 % CI)	Odds ratio (95 % CI)	Odds ratio (95 % CI)	Odds ratio (95 % CI)
Body mass index				
Poor	1 (Ref.)	1 (Ref.)	1 (Ref.)	1 (Ref.)
Intermediate	0.86(0.72-0.98)	0.89(0.72-1.03)	0.83(0.66-1.02)	0.88(0.76-1.02)
Ideal	0.75(0.58-0.88)	0.72(0.64-0.82)	0.76(0.65-0.96)	0.98(0.92-1.20)
Physical activity				
Poor	1 (Ref.)	1 (Ref.)	1 (Ref.)	1 (Ref.)
Intermediate	0.62(0.43-0.85)	0.92(0.76-1.03)	0.82(0.74-0.92)	0.92(0.86-1.04)
Ideal	0.48(0.36-0.52)	0.82(0.72-0.96)	0.46(0.32-0.56)	1.12(0.96-1.22)
Diet				
Poor	1 (Ref.)	1 (Ref.)	1 (Ref.)	1 (Ref.)
Intermediate	1.03(0.89-1.24)	0.86(0.78-0.96)	0.82(0.72-0.96)	0.82(0.72-0.94)
Ideal	1.16(1.02-1.34)	0.46(0.16-1.26)	0.39(0.22-1.18)	0.81(0.25-4.38)
Total cholesterol				
Poor	1 (Ref.)	1 (Ref.)	1 (Ref.)	1 (Ref.)
Intermediate	0.89(0.72-1.06)	0.95(0.86-1.08)	1.06(0.98-1.22)	0.95(0.83-1.06)
Ideal	0.85(0.65-1.02)	1.13(0.96-1.26)	1.18(1.05-1.32)	1.16(0.96-1.24)
Blood pressure				
Poor	1 (Ref.)	1 (Ref.)	1 (Ref.)	1 (Ref.)
Intermediate	0.75(0.68-0.88)	0.87(0.72-1.02)	0.86(0.68-0.92)	0.86(0.68-0.92)
Ideal	0.72(0.64-0.83)	0.83(0.76-0.95)	0.76(0.70-1.08)	0.94(0.82-1.10)
Fasting blood glucose				
Poor	1 (Ref.)	1 (Ref.)	1 (Ref.)	1 (Ref.)
Intermediate	0.76(0.62-0.83)	0.72(0.62-0.88)	0.88(0.62-1.08)	0.72(0.62-0.90)
Ideal	0.48(0.43-0.58)	0.32(0.28-0.42)	0.82(0.65-1.02)	0.56(0.48-0.72)

^a^Odds ratios are adjusted for age

Table 3 shows the odds ratios (95 % CIs) of ideal Life’s Simple 7 scores for each individual reproductive risk factor after adjustment for age. Participants with earlier age at menarche, earlier age at menopause, high parity, and with history of pregnancy losses, had an increasingly lower odds of obtaining higher ideal Life’s Simple 7 scores (all *P* < 0.05).

**Table 3:** Odds ratios for ideal Life’s Simple 7 scores associated with individual reproductive risk factors among CSWHSP 2013–2014 participants^a^

Ideal life’s simple 7 scores	Earlier age at menarche	Earlier age at menopause	With more than three children	Pregnancy losses
	Odds ratio (95 % CI)	Odds ratio (95 % CI)	Odds ratio (95 % CI)	Odds ratio (95 % CI)
0-1	1 (Ref.)	1 (Ref.)	1 (Ref.)	1 (Ref.)
2	0.66(0.56-0.76)	0.78(0.62-0.82)	0.72(0.62-0.82)	1.02(0.92-1.18)
3	0.62(0.55-0.72)	0.72(0.66-0.86)	0.66(0.58-0.76)	0.96(0.82-1.12)
4	0.58(0.52-0.68)	0.68(0.54-0.72)	0.62(0.52-0.68)	0.92(0.78-1.08)
5	0.46(0.36-0.52)	0.58(0.52-0.66)	0.56(0.48-0.66)	0.82(0.72-0.96)
6-7	0.32(0.22-0.38)	0.46(0.32-0.58)	0.42(0.38-0.56)	0.76(0.66-0.92)
*P* value	<0.001	<0.001	<0.001	0.025

^a^Odds ratios are adjusted for age

Table 4 shows odds ratios (95 % CIs) for ideal Life’s Simple 7 scores associated with exposure to cumulative reproductive risk after adjustment for age. Participants with a greater number of reproductive risk factors were significantly less likely to have higher ideal Life’s Simple 7 scores.

**Table 4:** Odds ratios for ideal Life’s Simple 7 scores associated with cumulative reproductive risk score among CSWHSP 2013–2014 participants^a^

Ideal life's simple 7 scores	Number of reproductive risk factors		
	1	2	3 or 4
0-1(Ref.)	1 (Ref.)	1 (Ref.)	1 (Ref.)
2	0.86(0.76-1.02)	0.74(0.56-0.88)	0.76(0.64-0.92)
3	0.78(0.68-0.96)	0.68(0.58-0.83)	0.72(0.56-0.88)
4	0.72(0.62-0.88)	0.62(0.54-0.76)	0.64(0.52-0.78)
5	0.65(0.48-0.75)	0.42(0.36-0.56)	0.56(0.46-0.68)
6-7	0.52(0.42-0.66)	0.22(0.16-0.26)	0.16(0.12-0.22)

The reference group consists of participants with no reproductive risk factors

^a^Odds ratios are adjusted for age

## Discussion

Our results demonstrated that Chinese postmenopausal women who had greater number of reproductive risk factors were less likely to have ideal levels of several components of the American Heart Association’s Life’s Simple 7. As exposure to the number of reproductive risk factors increased, individuals were less likely to have ideal levels of cardiovascular health behaviors and factors assessed using the Life’s Simple 7 metric. These findings highlight the importance of accounting for multiple reproductive risk factors in examining associations between reproductive history and ideal cardiovascular health in women after menopause.

Female-specific factors, primarily using single indicators of reproductive history, have been previously shown to be associated with differences in the presence of CVD risk factors [15–21]. In a population-based cross-sectional study from Korea, Jung et al. demonstrated that women with shorter ovarian hormone exposure defined as duration between menarche and menopause had increased cardiovascular risk [15]. Using data from the Cardiovascular Risk in Young Finns study, Skilton et al. showed that the progression of carotid atherosclerosis over a 6-year period is increased in females who gave birth during the same period, independent of traditional risk factors [20]. Similarly, using population-based cross-sectional data from China, Feng et al. demonstrated that age at menarche, reproductive years, and menopause status were significantly associated with body composition, insulin sensitivity and blood lipid levels [21]. However, reproductive risk factors developing in different stages throughout a woman’s life are seldom singular experiences and the present study has demonstrated that cumulative reproductive risk measures may better represent unfavorable reproductive profile than do single indicators. Furthermore, to the best of our knowledge, there are few previous studies on the association of reproductive factors and cardiovascular health metrics. Further cross-ethnic research is needed to clarify whether the association between cumulative reproductive risk exposure and ideal cardiovascular health is established among women.

This major strength of the study was its relatively large sample paired with first investigation of cumulative reproductive risk and ideal cardiovascular health. However, this study has several limitations. First, cross-sectional nature of the study should be considered when interpreting the findings reported. Second, Selection bias might have occurred because participants were community residents comprising urban women from middle to upper socioeconomic strata who underwent a health screening in a single area of China. Third, since privacy considerations, the reproductive risk profile might be misclassified due to the lack of detailed information about the childbearing history. Thus the current findings are probably underestimates of the true magnitude of the association between cumulative reproductive risk and ideal cardiovascular health. Fourth, we assigned the same weight to the reproductive risk factors in statistical analysis, which may have simplified and misestimated the actual association between the cumulative reproductive risk score and Life’s Simple 7 scores. Finally, there also remained a possibility of unmeasured factors or residual confounding might attenuate the strong ORs estimates in this study.

With these strengths and limitations in mind, the present study is the first one to investigate the cumulative effect of reproductive factors on ideal cardiovascular health for Chinese postmenopausal women. Our findings have possible policy implications. It has been identified that endogenous sex hormones have strong links to CVD risk in postmenopausal women [22]. Reproductive history including age at menarche, age at menopause, and gravidity (parity and pregnancy losses) are associated with CVD risk factors, and may best be addressed through primordial prevention. In spite of the progress on evaluating and managing CVD risk in women and the significance of reproductive history in this evaluation [23, 24], gaps in knowledge remain about female-specific risk factors, diagnostic and treatment options that might reduce mortality from CVD and improve outcomes for women [25]. One reason for this might be that the history of reproductive events does not play a superior role in the cardiovascular risk profile of postmenopausal women and most of the burden of CVD is by means of the traditional risk factors [24]. Programs that improve current risk assessment strategies by identifying female-specific risk factors during reproductive life, screen women affected by one or more reproductive manifestations of CVD risk, best implement current risk management strategies in these women to offer professional support can be instrumental to prevention of CVD. While this is routinely unappreciated in clinical practice, awareness several reproductive factors can influence ideal cardiovascular health may help clinicians and public health professionals further identify high risk women and thus develop and implement more effective lifestyle management plans [26, 27]. Moreover, since women play influential role in families, motivating midlife women in facing the challenge of adopting healthier behaviors may reap significant and immediate benefits in reducing the risks and consequences of CVD for the health and well being of women and their families [28]. Hence, this issue must be addressed at the government, community and individual levels in order to help them overcome CVD risk and improve their overall health after menopause.

## Conclusions

Findings of this study suggest that achieving ideal levels of several cardiovascular health behaviors and factors was less likely in postmenopausal Chinese women with relative short-term exposure to estrogen. In addition, as the exposure to the number of reproductive risk factors increased, women were increasingly less likely to achieve ideal levels of cardiovascular health after menopause. These findings highlight the need for sufficient resources and appropriate approaches to address multiple reproductive risk exposure among women out the protective effect of estrogen. To evaluate more precisely the effect of the reproductive history on the cardiovascular health for postmenopausal women, large cohort study of postmenopausal women would have to be conducted, using a long follow-up period as well as reliable information regarding the major reproductive factors.
